# 

**Published:** 1895-01

**Authors:** 


### CONTENTS.

CLINICAL LECTURE—

1. Chronic Bronchitis. 2. Endocarditis. By R. C. M. Page, M.D., New York... 641

ORIGINAL COMMUNICATIONS—

Management of the New Born. By W. Z. Holliday, M.D., Augusta, Ga.......... 647
Migraine. By Hugh Hagan, M.D., Atlanta, Ga.......... 656
The Parenchymatous Rupture Treatment. By C. Arnold F. Lindorme, Ph.D., M.D., Atlanta, Ga.......... 664

SOCIETY REPORTS—

Augusta Academy of Medicine.......... 667

CORRESPONDENCE—

Psoas Abscess.......... 673
The Craig Colony for Epileptics.......... 674

EDITORIAL—

The Medical Law.......... 676
More About the Serum Therapy.......... 679
The Atlanta Society of Medicine.......... 681

SELECTIONS AND ABSTRACTS—

Skin Diseases and Syphilis.......... 682
General Medicine and Therapeutics.......... 687
Obstetrics and Gynecology.......... 693

MEDICAL ITEMS.......... 698
BOOK REVIEWS.......... 700
ADVERTISERS' INDEX TO ADVERTISEMENTS.......... xii
MEDICAL ITEMS—Continued.......... vi, ix, x, xi

